# CAREGIVERS: PURPOSEFUL AGING LOS ANGELES (PALA) SURVEY OF CITY AND COUNTY EMPLOYEES

**DOI:** 10.1093/geroni/igz038.3553

**Published:** 2019-11-08

**Authors:** Iris Aguilar, Donald A Lloyd, Laura Trejo, Maria P Aranda

**Affiliations:** 1 USC Edward R. Roybal Institute on Aging, Los Angeles, California, United States; 2 Los Angeles Department of Aging, Los Angeles, California, United States; 3 University of Southern California, Los Angeles, California, United States

## Abstract

The City and County of Los Angeles Age-Friendly Cities initiative, Purposeful Aging Los Angeles (PALA), 2017 needs assessment included an online survey of the workforce expected to implement age friendly policies and programs. The study aim was to describe the rate and characteristics of family caregivers within in the City and County workforce (employees). The overall response rate was 6.5% (City 4.5%, County 7.5%) for a final sample size of 9,071. The family caregiver category was derived from the following survey questions: 1) Are you or someone else in your home caring for a family member or friends who are experiencing problems/thinking that is affecting their ability to work or live a normal life? and 2) Are you a family caregiver? The analysis revealed that 34% of all respondents were family caregivers and of those, 53% cared for adults and 12% cared for children under the age of 18 (minors). Of the 53% caregiving for adults, 40% cared for someone with memory/thinking problems including but not limited to dementia (cognitive impairment). 15% of caregivers of minors were also taking care of an adult with cognitive impairment. The current caregiving research literature suggests that family caregivers experience higher rates of depression, anxiety, and social isolation due to the caregiving demands. Caregiving demands have the potential to create work-family conflicts that are important for employers to consider when designing wellness programs, and other supportive programs. It is critical to identify ways to address caregiving implications on social services, healthcare, and the environment.

